# Tumor virus–induced lineage survival circuit drives Merkel cell carcinogenesis

**DOI:** 10.1172/JCI200581

**Published:** 2025-12-15

**Authors:** Masahiro Shuda

**Affiliations:** Cancer Virology Program, University of Pittsburgh Medical Center, Hillman Cancer Center, Pittsburgh, Pennsylvania, USA.

## Abstract

Approximately 80% of Merkel cell carcinoma (MCC) cases are caused by Merkel cell polyomavirus (MCV), driven by its T antigen oncogene. Why MCV drives MCC, a skin cancer that displays the neuroendocrine Merkel cell phenotype, remains unclear. In this issue of the *JCI*, Miao et al. demonstrated that MCC tumor survival requires neuroendocrine-lineage transcription factors, which are recruited to superenhancers (SEs) with the viral small T antigen oncoprotein to promote the neuroendocrine Merkel cell lineage of the cancer. Surprisingly, SEs mapped near the MCV integration site in MCC, and two SE-associated neuroendocrine transcription factors drove viral T antigen gene expression. MCV oncogene and neuroendocrine transcriptional network interactions rendered this viral tumorigenesis dependent on the Merkel cell lineage. Together with reports from other groups, the findings explain why MCV-associated cancer is specifically linked to the Merkel cell phenotype and identify epigenetic strategies targeting of lineage-dependent oncogene circuitry to treat virus-positive MCC.

## MCV oncogene promotes tumorigenesis and Merkel cell phenotype

Merkel cell polyomavirus (MCV) is a small, circular, double-stranded DNA virus discovered from human Merkel cell carcinoma (MCC), an aggressive skin cancer (1). MCV is ubiquitous in the general population and chronically shed from human skin, where it establishes a lifelong infection in unknown skin cells. In immunocompromised populations, however, MCV is a cause of MCC. MCC tumor cells display a phenotype similar to that of tactile sensor Merkel cells, a neuroendocrine lineage present in the epidermis.

The majority of MCCs (~80%) harbor clonally integrated MCV genomes that express the viral T antigen gene. The T antigen gene produces two viral proteins: the small T (ST) protein and the large T (LT) protein, the latter having C-terminal DNA helicase truncations. Consistent with MCV’s etiological role in MCC, MCV-positive MCC tumor cells exhibit oncogenic dependence on viral gene expression and require both ST and LT expression for tumor growth. MCV ST is a major oncoprotein capable of inducing oncogenic transformation in NIH3T3 and Rat1 rodent fibroblast cells. Whereas MCV LT protein promotes cell proliferation by binding to Rb family tumor suppressor proteins through its well-conserved binding domain, MCV ST promotes cell transformation through various other mechanisms. One of ST’s unique oncogenic properties in MCC is its ability to form the LMYC-MAX-EP400 transcription complex on chromatin, which activates LMYC-mediated oncogenic transcription (2).

In addition to oncogenic cell proliferation, T antigen oncoproteins promote Merkel cell signature genes such as cytokeratin 20, a marker exploited for MCC diagnosis, as well as genes encoding transcription factors such as ATOH1 and SOX2 that are important for normal Merkel cell development (3). SOX2 is the T antigen downstream cellular oncogene essential for MCC proliferation and directly regulates ATOH1 gene expression. In cocultures of MCC cells and keratinocytes, inhibition of the SOX2/ATOH1 pathway ablates MCC proliferation and induces the cellular quiescence associated with neuronal differentiation in a manner similar to T antigen inhibition (3). This observation is also consistent with the hypothesis that MCV does not transform normal Merkel cells into MCC. Instead, MCV promotes a Merkel cell phenotype in cells of unknown origin during T antigen–induced transformation.

## Merkel cell lineage addiction of MCV-transformed MCC

In this issue of the *JCI*, Miao et al. identified and characterized the superenhancer (SE) element in MCC that is maintained by Merkel cell/neuroendocrine lineage transcription factors, including INSM1, ISL1, LHX3, POU4F3, SOX2, and ATOH1 and the MCV ST-LMYC-MAX-EP400 transcriptional activator complex (4). This genetic element was revealed to be a hub that activated oncogenic and Merkel/neuroendocrine gene expression programs. Surprisingly, the MCC SE communicated with the integrated viral genome. Miao and colleagues observed that SEs mapped on the MCC tumor genome are associated with MCV integration sites and that two of the SE occupants, ISL1 and POU4F3, drive transcription of the viral T antigen oncogene. This *cis*-regulatory circuit establishes a positive feedback loop that renders MCV T antigen expression dependent on the SE and promotes oncogenic gene expression through the ST transcription complex. Consequently, MCV-transformed tumors are forced to adopt a Merkel cell lineage–dependent gene expression program and manifest a Merkel cell phenotype.

## Epigenetic addiction as a therapeutic target for MCC

It was previously shown that MCC tumor growth is sensitive to inhibitors of various epigenetic enzymes (5, 6). In the present study, Miao and colleagues demonstrated that induction of histone hyperacetylation by histone deacetylase (HDAC) inhibitors disrupted the SE-dependent feedback loop on the chromosome and suppressed MCC tumor growth. Miao et al. nicely discussed this mechanism by comparing the catastrophic consequences of overclocked genes driven by SEs to an overclocked CPU, with both scenarios requiring a mechanism to “cool down.” They showed that suppression of the HDAC that cools down SE activity induced topological blurring by inducing genome-wide histone acetylation, thereby disrupting feed-forward looping of SEs that are essential for activating target MCC oncogenes. Their data highlight an epigenetic vulnerability that can be exploited for precision therapy using epigenetic targeting of SE-mediated oncogene dependencies in MCC.

This approach could also be applicable to nonviral cancers. Microphthalmia-associated transcription factor (MITF) is a similar lineage-dependent oncogene that is amplified in non-viral skin cancer melanoma (7). MITF is required for melanocytic oncogenesis and was found to be one of the SE occupants in melanoma (8). HDAC inhibition has been shown to decrease MITF and suppress melanoma growth (9), possibly through the disruption of SE looping. Regardless of the etiology, targeting of SEs by HDAC inhibition could be a promising therapeutic strategy for lineage-addicted cancers.

## Remaining questions related to initiation of MCC

The results of Miao et al. support the hypothesis that MCC may not arise from Merkel cells. Rather, MCC enforces a Merkel cell/neuroendocrine lineage phenotype commitment during viral transformation. Although Merkel cells were previously thought to be the origin of MCC, ex vivo MCV infection experiments demonstrated dermal fibroblasts to be the primary MCV target cells, whereas no MCV infection was confirmed in Merkel cells (10). Normal Merkel cells appear to be resistant to MCV-mediated transformation, as shown by a transgenic mouse (11). Therefore, although MCV efficiently infects dermal fibroblasts, other skin cells can also be infected with MCV and transformed into MCC.

It is likely that MCV transforms Merkel progenitor cells in the skin, since the transcription factors required for the viral T antigen to establish the oncogenic circuit become available at some point during the differentiation process of these cells after MCV infection (12). Recent studies highlight hair follicle stem cells as a candidate. Trichoblastoma, a benign tumor that arises from the hair follicle’s germinal epidermis, is suggested to give rise to MCC by MCV infection (13). SOX9-positive hair follicle stem cells can give rise to Merkel cells in postnatal mouse skin, and expression of viral ST combined with RB1 knockout can reprogram the epithelial stem cells into neuroendocrine tumors (14). The successful development and use of mouse MCC tumor models revealed that transgenic expression of ATOH1, along with viral ST and/or LT in basal epithelial cells, gives rise to MCC-like tumors from the hair follicle, suggesting that ATOH1, an essential lineage factor for Merkel cell development (15), is critical for MCV-induced MCC (16). Other recent studies suggest that ATOH1 is a critical factor for MCC tumorigenesis (3, 5). Determining how the lineage transcription factors, including ATOH1, become epigenetically accessible in unknown MCV target cells and available for the viral T antigen to regulate their transcription would further help us understand the initiation of MCV-induced tumorigenesis.

Before the discovery of MCV, the molecular basis of MCC was unknown despite the urgency imbued by its high mortality rate (17). We look forward to learning more about the origin cells of MCC that are targeted by MCV, the development of a more effective therapeutic strategy, and the mechanism underlying the approximately 20% of cases of nonviral MCC.

## Funding support

This work is the result of NIH funding, in whole or in part, and is subject to the NIH Public Access Policy. Through acceptance of this federal funding, the NIH has been given a right to make the work publicly available in PubMed Central.

- NIH grant R01AI181892.

## Version 1 12/15/2025

Electronic publication
